# Supplementary material for: Genetic associations for two biological age measures point to distinct aging phenotypes
Source: Aging Cell. 2021 May 26;20(6):e13376. doi: 10.1111/acel.13376 (PMC8208797; doi:10.1111/acel.13376)

Figure S1 Quantile-Quantile plots for BioAgeAccel (genomic inflation factor lambda 1.11) and PhenoAgeAccel (lambda 1.12) (expected distributions and 95% confidence intervals depicted in red dash lines and blue shades)

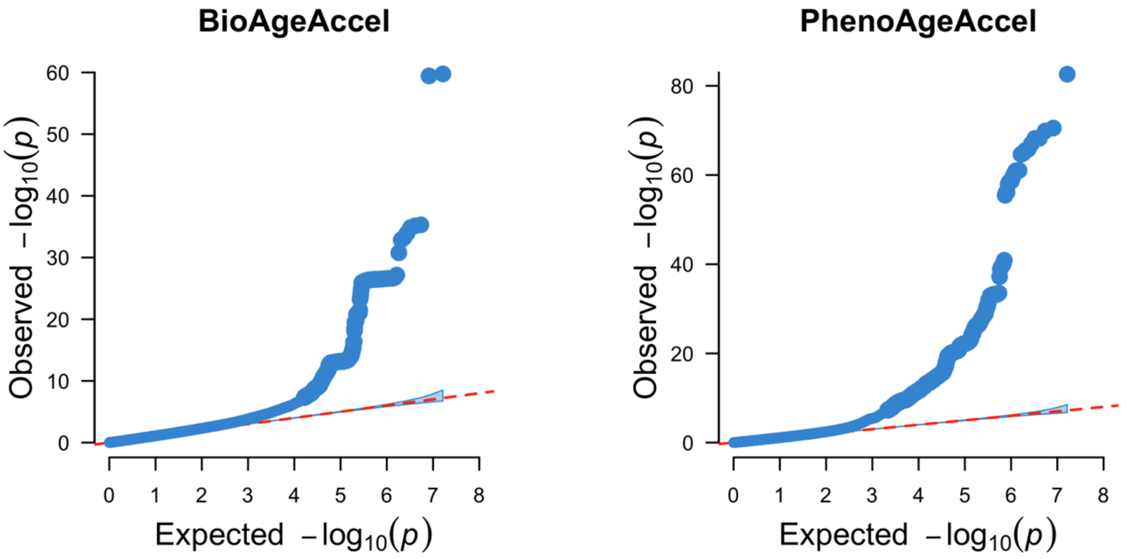

Figure S2 Genetic correlations between PhenoAgeAccel or BioAgeAccel and its elements

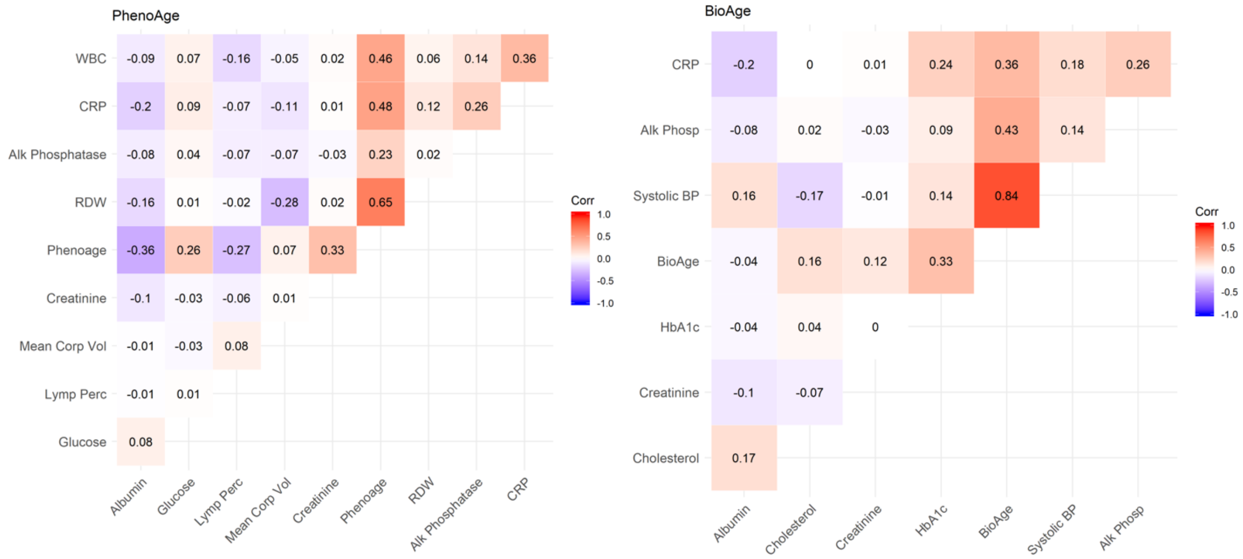

Figure S3 Genetic correlations between PhenoAgeAccel or BioAgeAccel and diseases or parental mortality

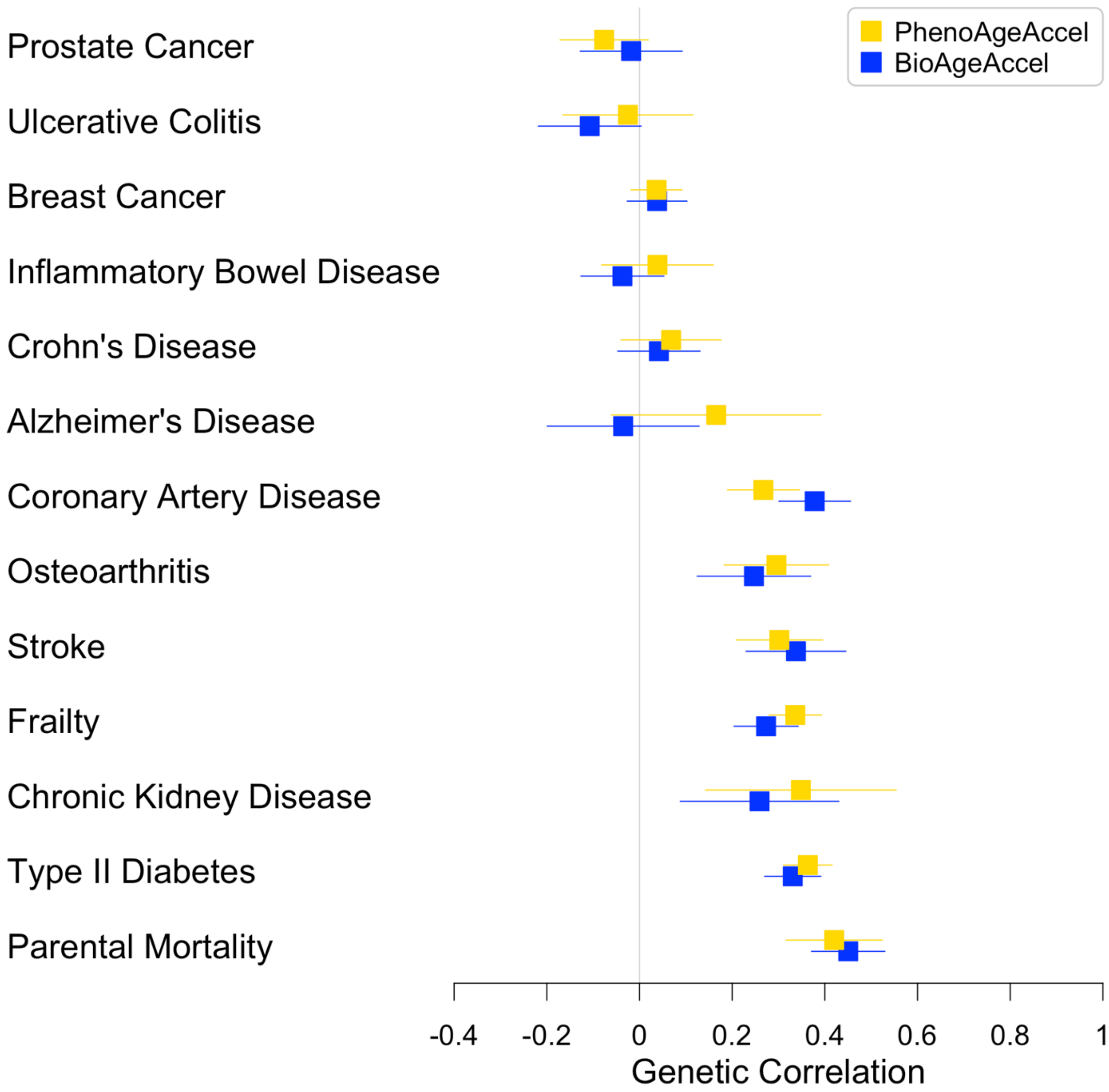

Figure S4 Genetic correlations between PhenoAgeAccel or BioAgeAccel and physical measures

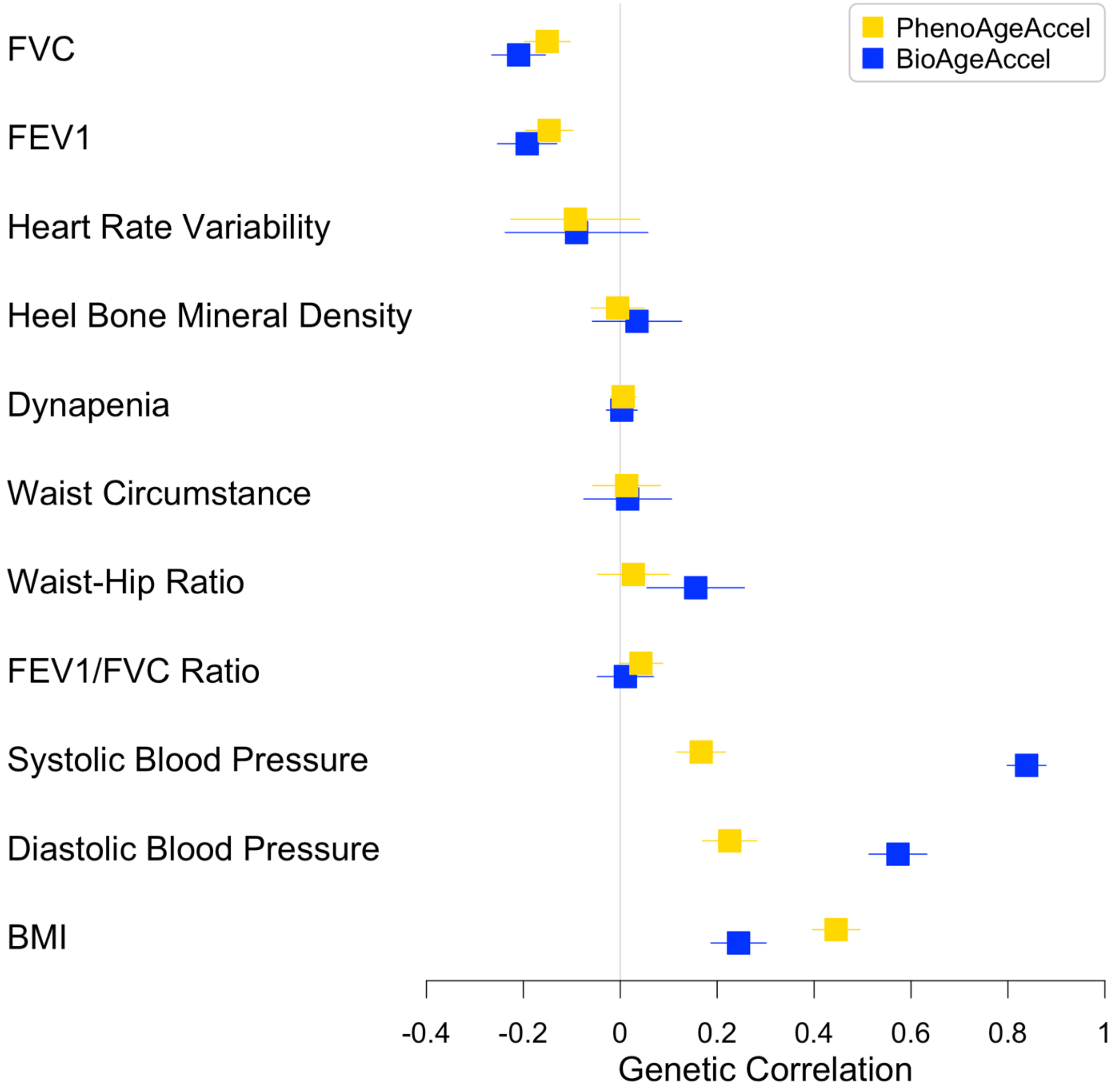

Figure S5 Genetic correlations between PhenoAgeAccel or BioAgeAccel and hematology traits

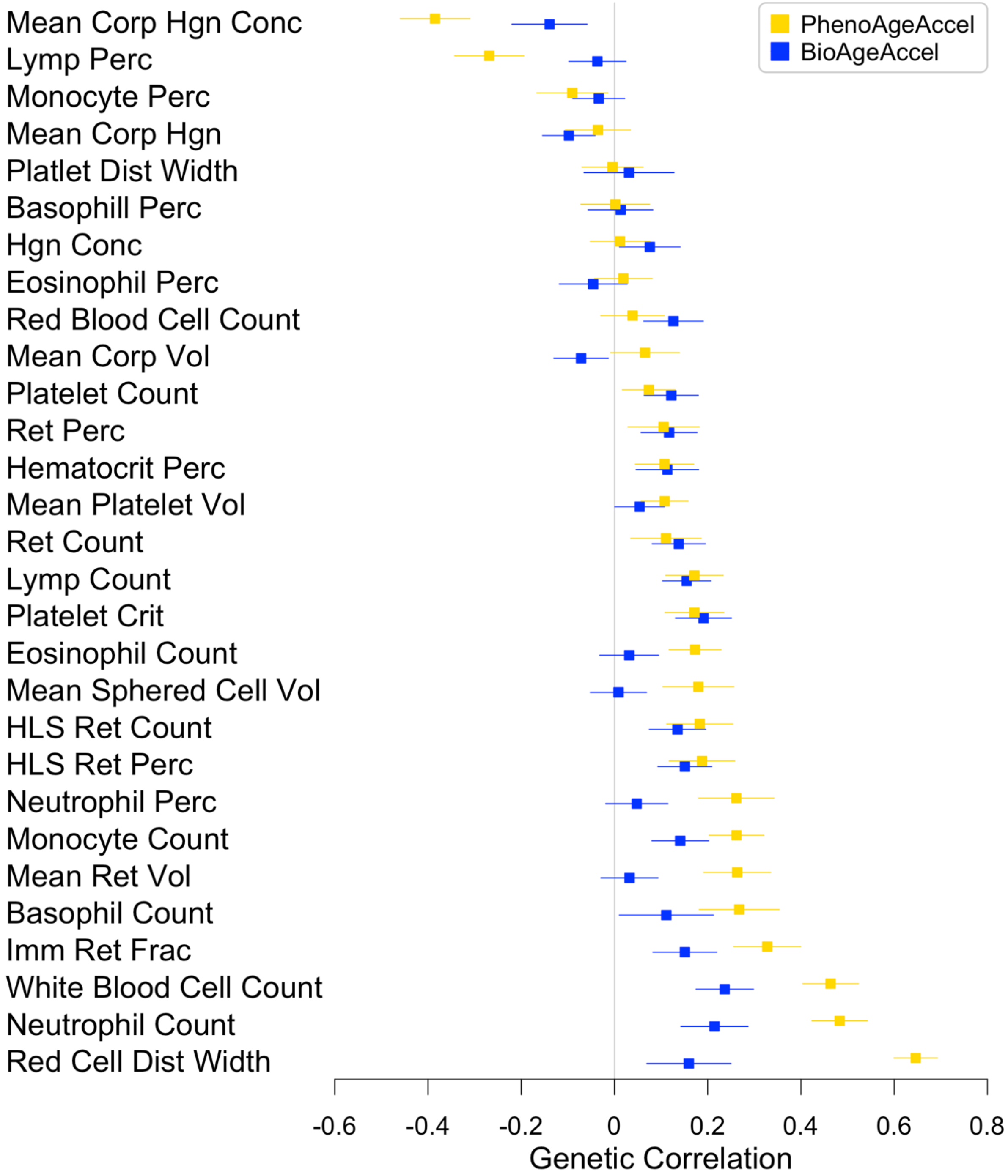

Mean Corp Hgn Conc: mean corpuscular hemoglobin concentration;
Lymp Perc: lymphocyte percentage;
Hgn Conc: hemoglobin concentration;
Ret Perc: reticulocyte percentage;
Mean Platelet Vol: mean platelet volume;
HLS Ret Count: high light scatter reticulocyte count;
Imm Ret Frac: immature reticulocyte fraction

Figure S6 Genetic correlations between PhenoAgeAccel or BioAgeAccel and biomarkers

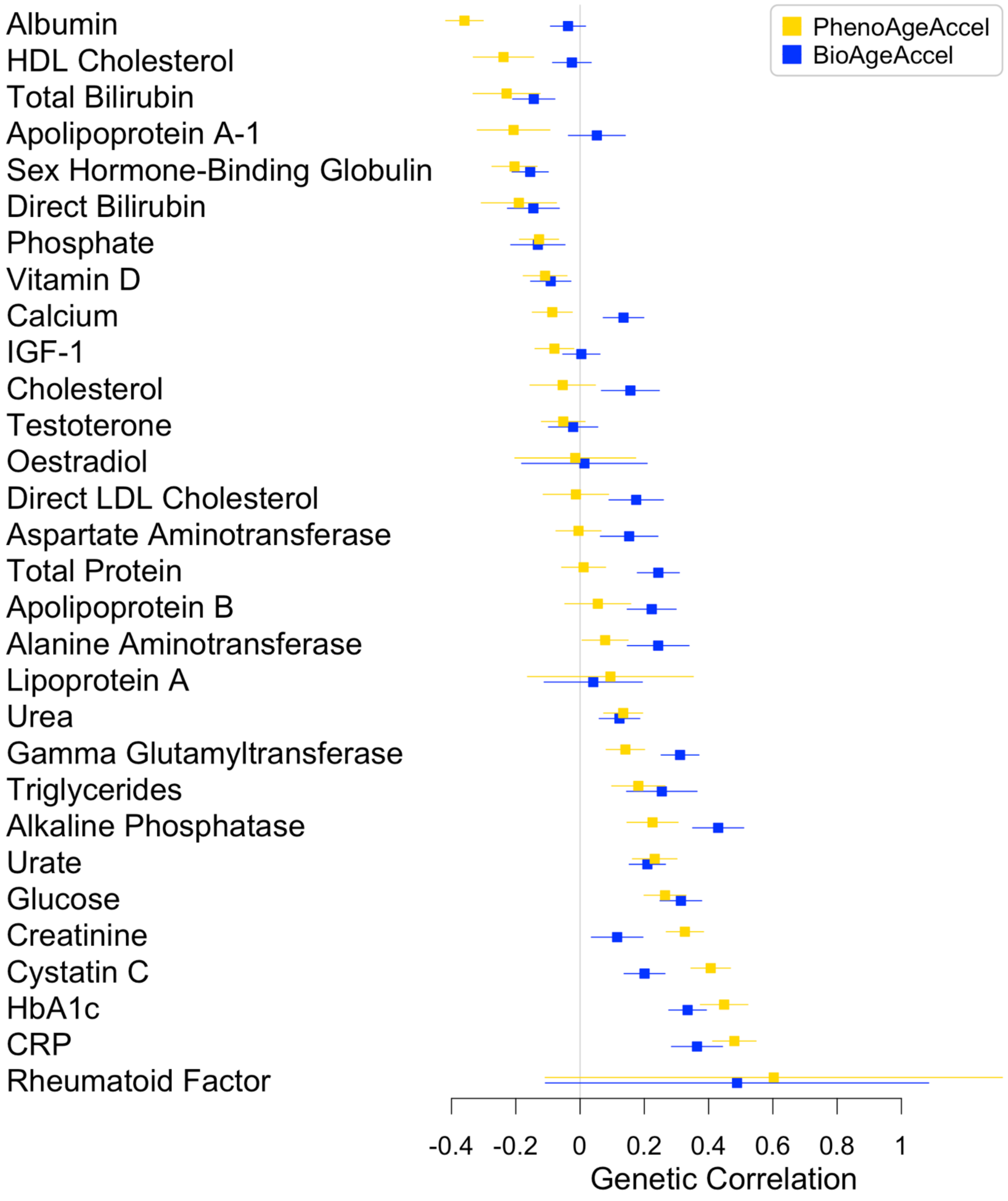

Figure S7 Mean standard deviation (SD) differences in levels of biomarkers between the top 20% or 40-60% and the bottom 20% of PhenoAgeAccel (in red) or BioAgeAccel (in blue) polygenic risk score (PRS) (*significantly associated with the top 20% of PhenoAgeAccel PRS at the 5% false-discovery-rate adjusted level; +significantly associated with the top 20% of BioAgeAccel PRS at the 5% false-discovery-rate adjusted level)

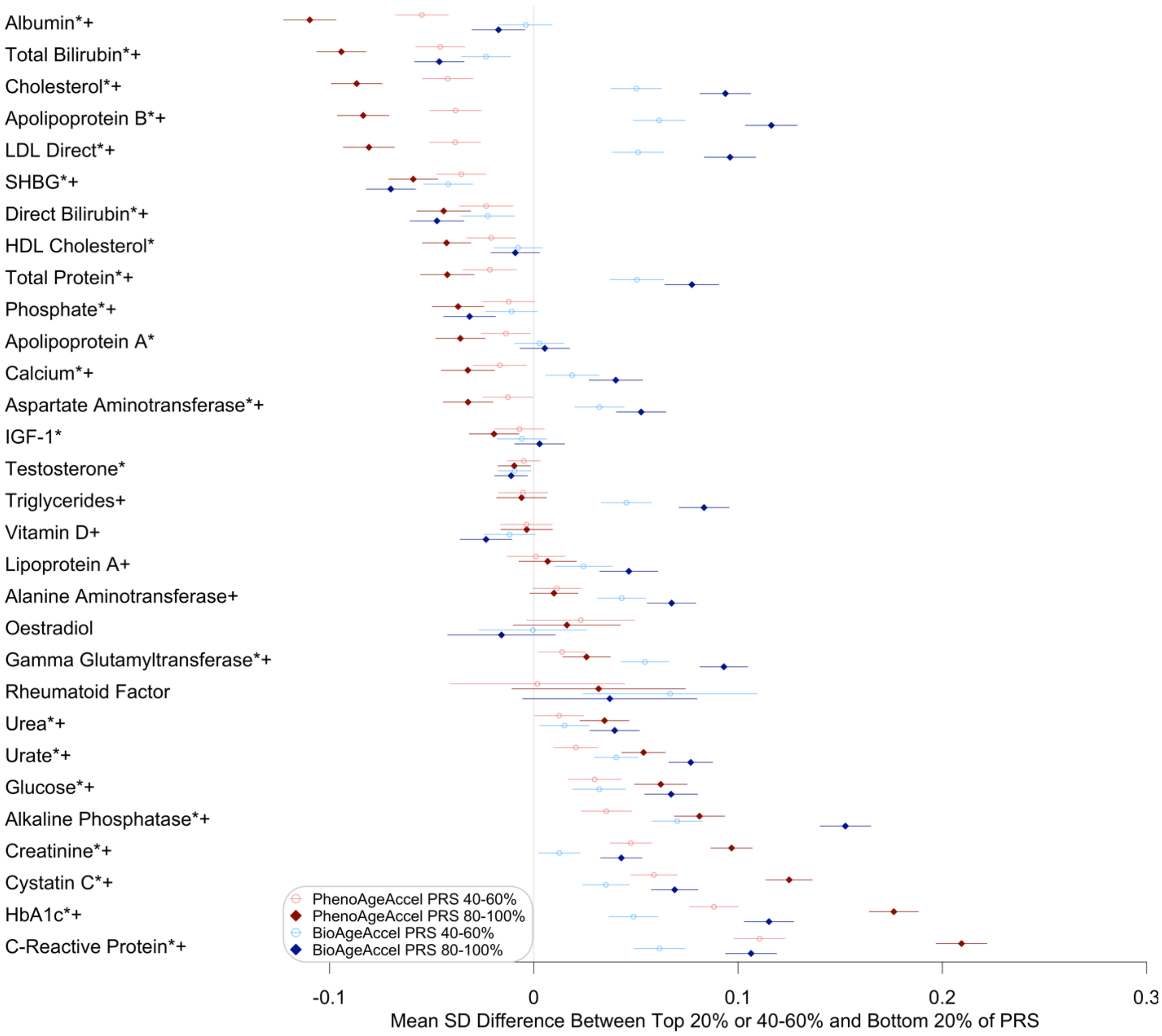

Figure S8 Mean standard deviation (SD) differences in levels of blood counts between the top 20% or 40-60% and the bottom 20% of PhenoAgeAccel (in red) or BioAgeAccel (in blue) polygenic risk score (PRS) (*significantly associated with the top 20% of PhenoAgeAccel PRS at the 5% false-discovery-rate adjusted level; +significantly associated with the top 20% of BioAgeAccel PRS at the 5% false-discovery-rate adjusted level)

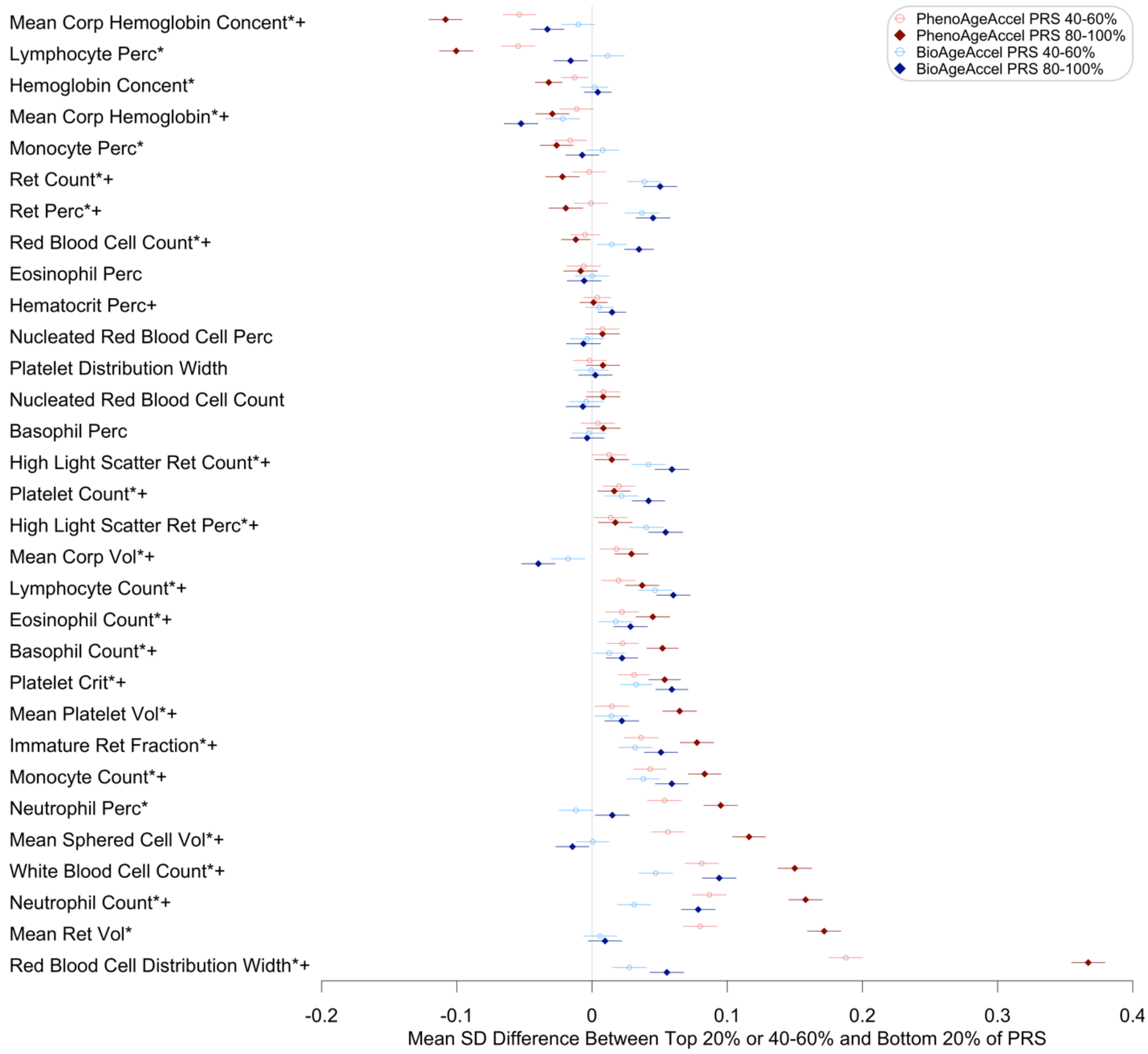

Supplement: Supplementary file 1 — Fig S1‐S8 [file ACEL-20-e13376-s001.pdf]
