# Supplementary material for: Genetic associations for two biological age measures point to distinct aging phenotypes
Source: Aging Cell. 2021 May 26;20(6):e13376. doi: 10.1111/acel.13376 (PMC8208797; doi:10.1111/acel.13376)
Supplement: Supplementary file 2 — Table S1‐S11 [file ACEL-20-e13376-s002.pdf]

Table S1 A summary for demographics and biomarkers in PhenoAge or BioAge in UKB

Variable	Frequency (%) or Mean ± SD
Demographics (n=379,703)	
Sex (=female)	204,736 (54%)
Age at recruitment (years)	56.74 ± 8.02
Current age (set at April 26, 2020[1]) (years)	68.22 ± 7.96
Follow-up years from baseline to April 26, 2020 (years)	11.49 ± 1.55
Dead (=yes)	23,060 (6%)
Age at death (years)	69.06 ± 7.21
PhenoAge (years) (n=321,652)	54.43 ± 9.56
Albumin (g/L)	45.25 ± 2.54
Creatinine (umol/L)	72.04 ± 14.16
log C-reactive protein (CRP) (mg/L)	0.33 ± 1.04
Alkaline phosphatase (U/L)	83.03 ± 22.59
Glucose (mmol/L)	5.08 ± 0.93
Lymphocyte percentage (%)	28.61 ± 7.03
Mean corpuscular volume (fL)	91.38 ± 4.10
Red blood cell distribution width (RDW)	13.45 ± 0.83
White blood cell count (10^9 cells/L)	6.87 ± 1.70
BioAge (years) (n=294,293)	56.16 ± 8.17
Albumin (g/dL)	4.53 ± 0.25
Creatinine (mg/dL)	0.72 ± 0.14
CRP (mg/dL)	0.25 ± 0.34
Alkaline phosphatase (U/L)	83.00 ± 22.56
HbA1c (%)	5.43 ± 0.50
Systolic blood pressure (mmHg)	139.92 ± 19.23
Total cholesterol (mg/dL)	220.78 ± 43.14

[1] last death occurred on April 26, 2020, in the data

Table S2 Demographic and biomarker summary statistics in the training and testing samples

Variable	Training (n=126,982)	Testing (n=252,721)
Sex (=female)	68,437 (54%)	136,299 (54%)
Age at recruitment (years)	56.75 ± 8.01	56.74 ± 8.02
Current age (set at April 26, 2020[1]) (years)	68.23 ± 7.96	68.22 ± 7.97
Follow-up years from baseline to April 26, 202	11.48 ± 1.55	11.49 ± 1.55
Dead (=yes)	7,717 (6%)	15,343 (6%)
Age at death (years)	68.98 ± 7.19	69.11 ± 7.22
PhenoAge (years)	54.45 ± 9.55	54.43 ± 9.57
Albumin (g/L)	45.24 ± 2.54	45.25 ± 2.53
Creatinine (umol/L)	72.02 ± 14.14	72.05 ± 14.18
log CRP (mg/L)	0.33 ± 1.05	0.32 ± 1.04
Alkaline phosphatase (U/L)	83.11 ± 22.61	83.00 ± 22.58
Glucose (mmol/L)	5.08 ± 0.92	5.08 ± 0.93
Lymphocyte percentage (%)	28.62 ± 7.02	28.61 ± 7.03
Mean corpuscular volume (fL)	91.37 ± 4.11	91.38 ± 4.09
Red blood cell distribution width	13.45 ± 0.84	13.45 ± 0.83
White blood cell count (10^9 cells/L)	6.87 ± 1.71	6.87 ± 1.70
BioAge (years)	56.15 ± 8.15	56.16 ± 8.17
Albumin (g/dL)	4.53 ± 0.25	4.53 ± 0.25
Creatinine (mg/dL)	0.72 ± 0.14	0.72 ± 0.14
CRP (mg/dL)	0.25 ± 0.34	0.25 ± 0.34
Alkaline phosphatase (U/L)	83.06 ± 22.57	82.98 ± 22.56
HbA1c (%)	5.43 ± 0.50	5.43 ± 0.50
Systolic blood pressure (mmHg)	139.95 ± 19.19	139.91 ± 19.25
Total cholesterol (mg/dL)	220.72 ± 43.21	220.81 ± 43.11

Table S3 PhenoAgeAccel GWAS lead SNPs

SNP	Chr	bp	refA	freq	bJ	bJ_se	pJ	mapped genes	context	traits
rs1801133	1	11856378	G	0.66	-0.13	0.022	1.28E-09	*MTHFR*	missense	folic acid measurement, homocysteine measurement, high altitude adaptation
rs12037222	1	40064961	G	0.77	-0.2	0.025	1.98E-15	*PABPC4 - HEYL*	regulatory_region	C-reactive protein measurement
rs1805096	1	66102257	G	0.63	0.2	0.021	1.01E-20	*LEPR*	synonymous	C-reactive protein measurement
rs4129267	1	154426264	C	0.59	0.16	0.021	6.73E-15	*IL6R*	intron	asthma, pulmonary function measurement, C-reactive protein measurement, ankylosing spondylitis, psoriasis, ulcerative colitis, Crohn's disease, sclerosing cholangitis, interleukin-6 receptor measurement, fibrinogen measurement, interleukin 6 receptor subunit alpha measurement
rs857685	1	158577109	A	0.74	-0.22	0.024	7.19E-20	-	-	-
rs77832441	1	159683814	G	1	0.97	0.175	2.39E-08	-	-	-
rs7553007	1	159698549	G	0.67	0.19	0.022	2.44E-17	*CRP - AL445528.1*	intergenic	C-reactive protein measurement
rs1419114	1	203652444	A	0.1	-0.23	0.035	1.82E-11	-	-	-
rs12239046	1	247601595	T	0.37	-0.12	0.021	1.40E-08	*NLRP3*	intron	myeloid white cell count, fibrinogen measurement, neutrophil count, neutrophil percentage of leukocytes, C-reactive protein measurement, leukocyte count, eosinophil count, basophil count, granulocyte count
rs3811444	1	248039451	C	0.66	0.18	0.022	2.61E-16	*TRIM58*	missense	mean corpuscular volume, fatty acid measurement, oleic acid measurement, platelet count, blood metabolite measurement, mean platelet volume, mean corpuscular hemoglobin concentration, red blood cell distribution width, reticulocyte count, leukocyte count, erythrocyte count, hemoglobin measurement
rs3856447	2	8750266	A	0.4	-0.14	0.021	8.14E-12	-	-	-
rs1260326	2	27730940	T	0.39	-0.13	0.021	2.29E-09	*GCKR*	missense	gout, blood metabolite measurement, amino acid measurement, inflammatory bowel disease, lean body mass, glomerular filtration rate, serum creatinine measurement, protein measurement, red blood cell distribution width, low density lipoprotein cholesterol measurement, lipid measurement, type II diabetes mellitus, coffee consumption measurement, platelet crit, granulocyte count, triglyceride measurement, leukocyte count, C-reactive protein, measurement, serum albumin measurement, body height, serum gamma-glutamyl transferase measurement, fasting blood glucose measurement, urate measurement, overweight body mass index status, resting heart rate, neutrophil count, basophil count, total cholesterol measurement, uric acid measurement, alcohol consumption measurement, lipoprotein-associated phospholipase A(2) measurement, Crohn's disease, eosinophil count, caffeine metabolite measurement, non-alcoholic fatty liver disease, gallstones, C-peptide measurement, blood protein measurement, serum alpha-1-antitrypsin measurement, myeloid white cell count, glucose measurement, sodium measurement, coffee consumption, cups of coffee per day measurement, physical activity, coronary artery calcification, mannose measurement, lactate measurement, platelet count, lymphocyte count, protein C measurement, reticulocyte count, glucose tolerance test, ankylosing spondylitis, psoriasis, ulcerative colitis, sclerosing cholangitis, Hypertriglyceridemia, alcohol use disorder measurement, total blood protein measurement, metabolite measurement, hematocrit, chronic kidney disease
rs6734238	2	113841030	A	0.6	-0.13	0.021	1.51E-10	*IL1F10 - RNU6-1180P*	regulatory_region	fibrinogen measurement, interleukin 1 receptor antagonist measurement, total cholesterol measurement, C-reactive protein measurement, leukocyte count
rs560887	2	169763148	T	0.3	-0.18	0.023	1.72E-15	*SPC25, G6PC2*	intron	glucose measurement, A1C measurement, fasting blood glucose measurement, birth weight, parental genotype effect measurement, metabolic syndrome, blood metabolite measurement, protein measurement, amino acid measurement, HOMA-B, coronary artery calcification, metabolite measurement, body mass index
rs34762726	3	49689210	G	0.71	0.18	0.023	2.95E-15	-	-	-
rs35188965	5	1104938	C	0.42	0.15	0.021	5.45E-13	*SLC12A7*	intron	red blood cell distribution width, mean corpuscular volume, platelet component distribution width, neutrophil count, basophil count, mean platelet volume, myeloid white cell count, erythrocyte count, eosinophil count, granulocyte count
rs16897620	5	67575344	A	0.76	-0.14	0.024	1.76E-08	-	-	-
rs12657096	5	72406312	T	0.15	-0.19	0.029	7.02E-11	-	-	-
rs2546147	5	127552590	G	0.26	-0.22	0.024	2.57E-20	-	-	-
rs3131622	6	31420500	T	0.55	-0.15	0.021	6.20E-13	-	-	-
rs9270664	6	32566149	G	0.36	-0.13	0.022	1.23E-09	-	-	-
rs7775698	6	135418635	C	0.74	0.16	0.024	9.60E-12	*HBS1L*	intron	mean corpuscular hemoglobin, total cholesterol measurement, mean corpuscular volume, hemoglobin measurement, mean corpuscular hemoglobin concentration, low density lipoprotein cholesterol measurement, erythrocyte count, platelet count, hematocrit, red blood cell distribution width
rs592423	6	139840693	A	0.45	0.14	0.021	3.38E-11	*AL592429.2*	intron	mean corpuscular hemoglobin, reticulocyte count, A1C measurement, erythrocyte count, adiponectin measurement, mean corpuscular volume
rs17321515	8	126486409	A	0.53	-0.14	0.021	4.08E-12	*AC091114.1*	intron	triglyceride measurement, total cholesterol measurement, low density lipoprotein cholesterol measurement
rs8176746	9	136131322	G	0.94	0.28	0.043	4.54E-11	*ABO*	missense	hemoglobin measurement, mean corpuscular hemoglobin concentration, mean corpuscular volume
rs10828724	10	25207403	A	0.63	0.14	0.021	8.32E-11	-	-	-
rs7908745	10	45953767	A	0.68	-0.14	0.022	1.31E-09	*MARCH8*	missense	reticulocyte count
rs74436700	10	46111895	G	0.98	-0.4	0.069	8.55E-09	-	-	-
rs16926246	10	71093392	C	0.87	-0.2	0.031	1.46E-10	*HK1*	intron	hematocrit, A1C measurement, hemoglobin measurement
rs11023922	11	16363658	C	0.92	-0.27	0.037	4.51E-13	-	-	-
rs174548	11	61571348	C	0.69	0.26	0.022	8.18E-31	*FADS1, FADS2*	5_prime_UTR	delta-6 desaturase measurement, blood metabolite measurement, dihomo-gamma-linolenic acid measurement, chronic kidney disease, serum metabolite measurement, high density lipoprotein cholesterol measurement, total cholesterol measurement, triglyceride measurement, albumin:globulin ratio measurement, basophil count, eosinophil count, phospholipid measurement, cis/trans-18:2 fatty acid measurement, trans fatty acid measurement, platelet count, low density lipoprotein cholesterol measurement, esterified cholesterol measurement
rs11606890	11	100471466	A	0.9	0.21	0.035	6.91E-10	-	-	-
rs964184	11	116648917	G	0.13	-0.17	0.03	3.28E-08	*ZPR1*	3_prime_UTR	reticulocyte count, triglyceride measurement, Hypertriglyceridemia, low density lipoprotein cholesterol measurement, platelet component distribution width, vitamin K measurement, vitamin measurement, alpha-tocopherol measurement, coronary artery calcification, total cholesterol measurement, diastolic blood pressure, systolic blood pressure, hematocrit, ventricular rate measurement, glucose measurement, body mass index, high density lipoprotein cholesterol measurement, physical activity, coronary artery disease, response to high fat food intake, triglyceride change measurement, response to vitamin, diglyceride measurement, lipoprotein measurement, blood metabolite measurement, metabolic syndrome, vitamin E measurement, very long-chain saturated fatty acid measurement, large artery stroke, coronary heart disease, phospholipid measurement, mean corpuscular hemoglobin concentration, lipid or lipoprotein measurement, red blood cell distribution width, mean platelet volume, lipoprotein-associated phospholipase A(2) measurement, very low density lipoprotein cholesterol measurement, stroke, atrial fibrillation, cancer, heart failure, diabetes mellitus, mortality
rs2280503	12	51138687	A	0.66	-0.14	0.022	3.17E-11	-	-	-
rs79880068	12	54649978	C	0.9	0.21	0.034	3.70E-10	-	-	-
rs2393791	12	121423956	C	0.38	-0.15	0.021	7.45E-12	*HNF1A*	intron	serum gamma-glutamyl transferase measurement, C-reactive protein measurement
rs8013143	14	23494277	A	0.72	-0.17	0.023	4.66E-14	*PSMB5*	intron	reticulocyte count, red blood cell distribution width
rs230703	14	65267469	T	0.71	-0.14	0.023	6.38E-10	-	-	-
rs3169166	15	78563103	A	0.58	0.16	0.021	1.34E-14	*DNAJA4*	intron	reticulocyte count, red blood cell distribution width
rs78029804	15	91538920	C	0.87	-0.3	0.031	1.01E-22	-	-	-
rs8061637	16	228306	G	0.93	0.23	0.041	1.42E-08	-	-	-
rs12443881	16	28841777	C	0.6	-0.12	0.021	8.52E-09	-	-	-
rs17616063	16	51436882	A	0.92	0.25	0.039	7.02E-11	-	-	-
rs9939609	16	53820527	T	0.61	-0.16	0.021	1.62E-13	*FTO*	intron	type II diabetes mellitus, total cholesterol measurement, diastolic blood pressure, triglyceride measurement, systolic blood pressure, hematocrit, ventricular rate measurement, glucose measurement, body mass index, high density lipoprotein cholesterol measurement, stroke, coronary heart disease, atrial fibrillation, cancer, heart failure, diabetes mellitus, mortality, age at menarche
rs9914988	17	27183104	G	0.2	-0.14	0.026	3.20E-08	*ERAL1*	intron	reticulocyte count, A1C measurement
rs17781005	17	31132529	T	0.81	0.15	0.026	5.94E-09	-	-	-
rs8078723	17	38166879	T	0.61	-0.21	0.021	6.25E-23	*PSMD3 - AC090844.3*	regulatory_region	neutrophil count, leukocyte count
rs2292642	17	76395430	C	0.4	-0.12	0.021	6.59E-09	-	-	-
rs9944715	18	43831259	A	0.25	-0.16	0.024	6.09E-11	*C18orf25*	intron	red blood cell distribution width, mean corpuscular volume
rs1985157	19	18513594	T	0.59	-0.14	0.021	1.10E-10	*LRRC25 - SSBP4*	regulatory_region	granulocyte percentage of myeloid white cells, mosquito bite reaction itch intensity measurement
rs45512696	19	35550878	C	0.82	0.17	0.027	1.26E-09	-	-	-
rs429358	19	45411941	T	0.84	0.52	0.029	1.50E-72	*APOE*	missense	longevity, low density lipoprotein cholesterol measurement, Alzheimer's disease, amyloid-beta measurement, total cholesterol measurement, t-tau measurement, memory performance, Lewy body dementia, Lewy body dementia measurement, neuroimaging measurement, cognitive decline, health study participation, platelet count, parental longevity, neuritic plaque measurement, neurofibrillary tangles measurement, hippocampal volume, C-reactive protein measurement, red blood cell distribution width, cognitive impairment measurement, p-tau measurement, high density lipoprotein cholesterol measurement, mortality, beta-amyloid 1-42 measurement, waist-hip ratio, triglyceride measurement, hyperopia, cerebral amyloid angiopathy, physical activity measurement, hypertension, atrophic macular degeneration, age-related macular degeneration, wet macular degeneration, apolipoprotein E measurement, cerebral amyloid deposition measurement, vascular dementia
rs7412	19	45412079	C	0.92	-0.36	0.038	3.07E-21	*APOE*	missense	longevity, LDL cholesterol change measurement, total cholesterol measurement, lipoprotein-associated phospholipase A(2) measurement, acute coronary syndrome, coronary heart disease, high density lipoprotein cholesterol measurement, reticulocyte count, coronary artery disease, low density lipoprotein cholesterol measurement, response to statin, systolic blood pressure, lipoprotein A measurement, lipoprotein measurement, blood metabolite measurement, Alzheimer's disease, family history of Alzheimer’s disease,

SNP	Chr	bp	refA	freq	bJ	bJ_se	pJ	mapped genes	context	traits
										red blood cell distribution width, lipid measurement, response to darapladib, lipoprotein-associated phospholipase A(2) change measurement, late-onset Alzheimer's disease, apolipoprotein A 1 measurement, pulse pressure measurement, triglyceride measurement, clinical and behavioural ideal cardiovascular health
rs159428	20	31099311	T	0.47	-0.11	0.021	3.33E-08	-	-	-
rs2838701	21	46257269	G	0.87	-0.18	0.031	1.52E-09	-	-	-

Table S4 BioAgeAccel GWAS lead SNPs

SNP	Chr	bp	refA	freq	bJ	bJ_se	pJ	mapped genes	context	associated traits
rs17367504	1	11862778	A	0.84	0.07	0.012	9.03E-10	*MTHFR*	intron	diastolic blood pressure, mean arterial pressure, pulse pressure measurement, birth weight, parental genotype effect measurement, systolic blood pressure, smoking status measurement
rs149344982	1	21889760	G	0.99	0.25	0.038	6.48E-11	-	-	-
rs11591147	1	55505647	G	0.98	0.23	0.033	7.91E-13	*PCSK9*	missense	PCSK9 protein measurement, lipoprotein measurement, blood metabolite measurement, low density lipoprotein cholesterol measurement, total cholesterol measurement, alcohol consumption measurement, alcohol drinking, coronary artery disease, physical activity, osteoarthritis, knee, response to statin, LDL cholesterol change measurement
rs541041	2	21294975	G	0.18	-0.09	0.011	2.33E-14	*APOB - AC010872.2*	intergenic	low density lipoprotein cholesterol measurement, total cholesterol measurement, triglyceride measurement, response to statin
rs560887	2	169763148	T	0.3	-0.06	0.009	9.83E-11	*SPC25, G6PC2*	intron	fasting blood glucose measurement, metabolic syndrome, blood metabolite measurement, protein measurement, amino acid measurement, coronary artery calcification, A1C measurement, body mass index, glucose measurement, birth weight, parental genotype effect measurement, metabolite measurement, HOMA-B
rs16998073	4	81184341	A	0.71	-0.05	0.009	2.46E-08	*PRDM8 - FGF5*	intergenic	systolic blood pressure, pulse pressure measurement, diastolic blood pressure, mean arterial pressure, alcohol consumption measurement, glomerular filtration rate, hypertension
rs1173771	5	32815028	A	0.4	-0.05	0.009	6.19E-09	*NPR3 - AC025459.1*	regulatory_region	diastolic blood pressure, systolic blood pressure, smoking status measurement, pulse pressure measurement, mean arterial pressure, BMI-adjusted hip circumference, hypertension, body height
rs3130287	6	32050544	C	0.15	-0.08	0.012	4.54E-12	-	-	-
rs17477177	7	106411858	T	0.8	-0.09	0.011	4.62E-17	*AC004917.1 - LINC02577*	intergenic	pulse pressure measurement, smoking status measurement, systolic blood pressure
rs6601523	8	10635141	G	0.4	0.05	0.009	6.67E-09	-	-	-
rs17321515	8	126486409	A	0.53	0.06	0.009	2.20E-12	*AC091114.1*	intron	low density lipoprotein cholesterol measurement, triglyceride measurement, total cholesterol measurement
rs16926246	10	71093392	C	0.87	0.09	0.013	7.77E-13	*HK1*	intron	hemoglobin measurement, hematocrit, A1C measurement
rs2274224	10	96039597	G	0.57	0.05	0.009	2.41E-10	*PLCE1, PLCE1-AS1*	missense	body fat percentage, birth weight, parental genotype effect measurement
rs17249754	12	90060586	G	0.83	0.07	0.011	9.41E-09	*ATP2B1*	intron	diastolic blood pressure, mean arterial pressure, pulse pressure measurement, systolic blood pressure, hypertension, smoking status measurement
rs7497304	15	91429176	G	0.67	-0.05	0.009	1.89E-08	*FES*	intron	systolic blood pressure, alcohol drinking
rs77870048	16	69965021	C	0.95	-0.11	0.019	7.58E-09	-	-	-
rs55791371	19	11188153	A	0.88	0.14	0.013	4.95E-26	*SMARCA4*	intron	lipid measurement, coronary artery disease, myocardial infarction
rs58542926	19	19379549	C	0.92	0.11	0.016	1.78E-11	*AC138430.1, TM6SF2*	missense	triglyceride measurement, low density lipoprotein cholesterol measurement, physical activity, total cholesterol measurement, type II diabetes mellitus, vitamin measurement, alpha-tocopherol measurement
rs7412	19	45412079	C	0.92	0.26	0.016	3.16E-60	*APOE*	missense	triglyceride measurement, total cholesterol measurement, longevity, reticulocyte count, LDL cholesterol change measurement, response to statin, systolic blood pressure, coronary artery disease, family history of Alzheimer's disease, low density lipoprotein cholesterol measurement, high density lipoprotein cholesterol measurement, Alzheimer's disease, pulse pressure measurement, lipoprotein-associated phospholipase A(2) measurement, acute coronary syndrome, coronary heart disease, lipoprotein A measurement, apolipoprotein A 1 measurement, late-onset Alzheimers disease, lipoprotein measurement, blood metabolite measurement, red blood cell distribution width, clinical and behavioural ideal cardiovascular health, lipid measurement, response to darapladib, lipoprotein-associated phospholipase A(2) change measurement
rs1327235	20	10969030	A	0.52	-0.05	0.009	1.02E-08	*AL050403.2*	intron	systolic blood pressure, hypertension, diastolic blood pressure, smoking status measurement, mean arterial pressure

Table S5 A summary of aging traits in the testing set for PhenoAgeAccel and BioAgeAccel polygenic risk score analyses

Aging Trait	Category	Transformation	N	Mean ± SD or Frequency (%)
Alanine Aminotransferase	Biomarker	Inverse Normal	240,906	0 ± 1
Albumin	Biomarker	Inverse Normal	220,829	0 ± 1
Alkaline Phosphatase	Biomarker	Inverse Normal	241,011	0 ± 1
Apolipoprotein A	Biomarker	Inverse Normal	219,535	0 ± 1
Apolipoprotein B	Biomarker	Inverse Normal	239,822	0 ± 1
Aspartate Aminotransferase	Biomarker	Inverse Normal	240,112	0 ± 1
C-Reactive Protein	Biomarker	Inverse Normal	240,466	0 ± 1
Calcium	Biomarker	Inverse Normal	220,756	0 ± 1
Cholesterol	Biomarker	Inverse Normal	240,995	0 ± 1
Creatinine	Biomarker	Inverse Normal	240,874	0 ± 1
Cystatin C	Biomarker	Inverse Normal	240,976	0 ± 1
Direct Bilirubin	Biomarker	Inverse Normal	205,097	0 ± 1
Gamma Glutamyltransferase	Biomarker	Inverse Normal	240,875	0 ± 1
Glucose	Biomarker	Inverse Normal	220,591	0 ± 1
HbA1c	Biomarker	Inverse Normal	240,884	0 ± 1
HDL Cholesterol	Biomarker	Inverse Normal	220,739	0 ± 1
IGF-1	Biomarker	Inverse Normal	239,676	0 ± 1
LDL Direct	Biomarker	Inverse Normal	240,534	0 ± 1
Lipoprotein A	Biomarker	Inverse Normal	191,801	0 ± 1
Oestradiol	Biomarker	Inverse Normal	38,579	0 ± 1
Phosphate	Biomarker	Inverse Normal	220,416	0 ± 1
Rheumatoid Factor	Biomarker	Inverse Normal	21,395	0.01 ± 0.99
SHBG	Biomarker	Inverse Normal	218,774	0 ± 1
Testosterone	Biomarker	Inverse Normal	218,490	0 ± 1
Total Bilirubin	Biomarker	Inverse Normal	240,005	0 ± 1
Total Protein	Biomarker	Inverse Normal	220,610	0 ± 1
Triglycerides	Biomarker	Inverse Normal	240,794	0 ± 1
Urate	Biomarker	Inverse Normal	240,714	0 ± 1
Urea	Biomarker	Inverse Normal	240,832	0 ± 1
Vitamin D	Biomarker	Inverse Normal	230,540	0 ± 1
Basophil Count	Blood Count	Inverse Normal	244,759	0.03 ± 0.91
Basophil Perc	Blood Count	Inverse Normal	244,762	0 ± 1
Eosinophil Count	Blood Count	Inverse Normal	244,759	0 ± 0.99
Eosinophil Perc	Blood Count	Inverse Normal	244,762	0 ± 1
Hematocrit Perc	Blood Count	Inverse Normal	245,193	0 ± 1
Hemoglobin Concent	Blood Count	Inverse Normal	245,193	0 ± 1
High Light Scatter Ret Count	Blood Count	Inverse Normal	241,223	0 ± 1
High Light Scatter Ret Perc	Blood Count	Inverse Normal	241,224	0 ± 1
Immature Ret Fraction	Blood Count	Inverse Normal	241,223	0 ± 1
Lymphocyte Count	Blood Count	Inverse Normal	244,759	0 ± 1
Lymphocyte Perc	Blood Count	Inverse Normal	244,762	0 ± 1
Mean Corp Hemoglobin	Blood Count	Inverse Normal	245,191	0 ± 1
Mean Corp Hemoglobin Concent	Blood Count	Inverse Normal	245,189	0 ± 1
Mean Corp Vol	Blood Count	Inverse Normal	245,192	0 ± 1
Mean Platelet Vol	Blood Count	Inverse Normal	245,189	0 ± 1
Mean Ret Vol	Blood Count	Inverse Normal	241,223	0 ± 1
Mean Sphered Cell Vol	Blood Count	Inverse Normal	241,224	0 ± 1
Monocyte Count	Blood Count	Inverse Normal	244,759	0 ± 1
Monocyte Perc	Blood Count	Inverse Normal	244,762	0 ± 1
Neutrophil Count	Blood Count	Inverse Normal	244,759	0 ± 1
Neutrophil Perc	Blood Count	Inverse Normal	244,762	0 ± 1
Nucleated Red Blood Cell Count	Blood Count	Inverse Normal	244,754	0.01 ± 0.25
Nucleated Red Blood Cell Perc	Blood Count	Inverse Normal	244,752	0.01 ± 0.25
Platelet Count	Blood Count	Inverse Normal	245,192	0 ± 1
Platelet Crit	Blood Count	Inverse Normal	245,190	0 ± 1
Platelet Distribution Width	Blood Count	Inverse Normal	245,189	0 ± 1
Red Blood Cell Count	Blood Count	Inverse Normal	245,193	0 ± 1
Red Blood Cell Distribution Width	Blood Count	Inverse Normal	245,192	0 ± 1

Aging Trait	Category	Transformation	N	Mean ± SD or Frequency (%)
Ret Count	Blood Count	Inverse Normal	241,223	0 ± 1
Ret Perc	Blood Count	Inverse Normal	241,223	0 ± 1
White Blood Cell Count	Blood Count	Inverse Normal	245,191	0 ± 1
Bladder Cancer	Cancer		252,715	1702 (0.67%)
Colorectal Cancer	Cancer		252,715	3287 (1.3%)
Lung Cancer	Cancer		252,715	1775 (0.7%)
Melanoma Cancer	Cancer		252,715	2969 (1.17%)
Prostate Cancer	Cancer		116,418	4888 (4.2%)
Back Pain 3+ Months	Chronic Pain		251,717	43390 (17.24%)
Hip Pain 3+ Months	Chronic Pain		251,995	21839 (8.67%)
Knee Pain 3+ Months	Chronic Pain		251,854	41567 (16.5%)
Reaction Time	Cognitive	log	251,052	6.3 ± 0.19
Visual Memory Errors	Cognitive	log	252,489	1.44 ± 0.65
Age-Related Macular Degeneration	Disease		252,715	2028 (0.8%)
Anemia	Disease		252,715	7242 (2.87%)
Anxiety	Disease		252,715	8569 (3.39%)
Atrial Fibrillation	Disease		252,718	11481 (4.54%)
Breast Cancer	Disease		136,297	8901 (6.53%)
Chronic Kidney Disease	Disease		252,718	2745 (1.09%)
COPD	Disease		252,715	7723 (3.06%)
Coronary Artery Disease	Disease		252,715	23266 (9.21%)
Delirium	Disease		252,692	753 (0.3%)
Dementia	Disease		252,715	1158 (0.46%)
Depression	Disease		197,313	11002 (5.58%)
Heart Failure	Disease		252,715	4684 (1.85%)
Hypertension	Disease		252,715	82399 (32.61%)
Hypothyrodism	Disease		252,715	15482 (6.13%)
Kidney Cancer	Disease		252,715	895 (0.35%)
Liver Disease	Disease		252,718	4042 (1.6%)
Multiple Sclerosis	Disease		252,715	1118 (0.44%)
Osteoarthritis	Disease		252,715	26699 (10.56%)
Osteoporosis	Disease		252,715	7809 (3.09%)
Parkinson's Disease	Disease		252,715	1048 (0.41%)
Peripheral Vascular Disease	Disease		252,715	3531 (1.4%)
Pneumonia	Disease		252,715	10908 (4.32%)
Renal Failure	Disease		252,715	4212 (1.67%)
Rheumatoid Arthritis	Disease		252,715	4650 (1.84%)
Stroke	Disease		252,718	4720 (1.87%)
Type I Diabetes	Disease		252,715	1942 (0.77%)
Type II Diabetes	Disease		252,715	13900 (5.5%)
49-Item Frailty	Frailty	log	91,746	1.85 ± 0.54
Fried Frailty	Frailty		96,230	3516 (3.65%)
Parents' Attained Age	Lifespan		114,395	-0.02 ± 1.57
Both Parents Dead	Lifespan		216,116	114395 (53%)
Parents Both Top 10% Survival	Lifespan		47,969	4668 (9.73%)
Participant's Lifespan	Lifespan		10,242	67.35 ± 7.02
Participant Dead	Lifespan		252,718	10242 (4%)
BMI	Physical		251,890	27.37 ± 4.77
Diastolic Blood Pressure	Physical		235,946	82.17 ± 10.69
FEV1	Physical		163,274	2.87 ± 0.76
FEV1/FVC Ratio	Physical		163,274	0.76 ± 0.06
FVC	Physical		163,274	3.78 ± 0.96
Grip Strength	Physical		251,660	32.88 ± 11.31
Heel BMD	Physical		144,079	0.54 ± 0.14
Systolic Blood Pressure	Physical		235,939	139.9 ± 19.69

Table S6 A summary for demographics and biomarkers in PhenoAge or BioAge in HRS

Variable	Frequency (%) or Mean ± SD
PhenoAge (years) (n=5,572)	73.07 ± 13.85
Age in 2016	71.32 ± 9.86
Sex (=Femae)	3289 (59%)
2016 Albumin (g/L)	39.48 ± 3.08
2016 Creatinine (umol/L)	82.25 ± 26.12
2016 log C-reactive protein (CRP) (mg/L)	-1.46 ± 1.00
2016 Alkaline phosphatase (U/L)	80.53 ± 24.36
2016 Glucose (mmol/L)	6.12 ± 2.12
2016 Lymphocyte percentage (%)	28.54 ± 8.31
2016 Mean corpuscular volume (fL)	93.79 ± 5.49
2016 Red blood cell distribution width (RDW)	13.93 ± 1.27
2016 White blood cell count (10^9 cells/L)	6.76 ± 1.84
BioAge (years) (n=1,782)	71.06 ± 8.48
Age in 2016	73.84 ± 8.92
Sex (=Femae)	1055 (59%)
2016 Albumin (g/dL)	3.93 ± 0.31
2016 Creatinine (mg/dL)	0.94 ± 0.30
2016 CRP (mg/dL)	0.41 ± 0.70
2016 Alkaline phosphatase (U/L)	79.94 ± 23.38
2016 HbA1c (%)	5.62 ± 0.58
2016 Systolic blood pressure (mmHg)	127.98 ± 18.15
2016 Total cholesterol (mg/dL)	185.67 ± 41.39
BioAge (years) (n=4,909)	68.66 ± 9.51
2016 Age in 2016	71.24 ± 89.93
2016 Sex (=Femae)	2900 (59%)
2016 Albumin (g/dL)	3.95 ± 0.31
2016 Creatinine (mg/dL)	0.93 ± 0.29
2016 CRP (mg/dL)	0.41 ± 0.67
2016 Alkaline phosphatase (U/L)	80.12 ± 24.32
2016 + 2014 HbA1c (%)	5.57 ± 0.62
2016 + 2014 Systolic blood pressure (mmHg)	127.62 ± 18.59
2016 Total cholesterol (mg/dL)	187.06 ± 41.05

Table S7 Replication results in HRS for the PhenoAgeAccel lead SNPs from UKB

				US Health Retirement Study				UK Biobank				
Chr	bp	refA	rs	freq	b	se	p	freq	b	se	p	PVE (%)
19	45411941	T	rs429358	0.87	0.58	0.24	0.01	0.84	0.55	0.03	7.85E-83	0.34
19	45412079	C	rs7412	0.92	-0.38	0.30	0.20	0.92	-0.44	0.04	1.64E-31	0.13
11	61571348	C	rs174548	0.66	0.12	0.17	0.51	0.69	0.26	0.02	7.54E-31	0.12
17	38166879	T	rs8078723	0.63	-0.21	0.17	0.21	0.61	-0.21	0.02	6.93E-23	0.09
15	91538920	C	rs78029804	0.84	-0.11	0.22	0.61	0.87	-0.30	0.03	9.73E-23	0.09
1	66102257	G	rs1805096	0.60	0.23	0.17	0.17	0.63	0.20	0.02	9.79E-21	0.08
5	127552590	G	rs2546147	0.29	-0.38	0.18	0.03	0.26	-0.22	0.02	2.48E-20	0.08
1	158577109	A	rs857685	0.73	-0.19	0.18	0.29	0.74	-0.21	0.02	3.23E-19	0.07
1	159698549	G	rs7553007	0.66	0.27	0.17	0.10	0.67	0.18	0.02	2.34E-16	0.06
1	248039451	C	rs3811444	0.67	-0.19	0.17	0.27	0.66	0.18	0.02	3.72E-16	0.06
2	169763148	T	rs560887	0.26	-0.15	0.18	0.41	0.30	-0.18	0.02	1.69E-15	0.06
1	40064961	G	rs12037222	0.78	-0.02	0.20	0.92	0.77	-0.20	0.02	1.95E-15	0.06
3	49689210	G	rs34762726	0.72	0.20	0.18	0.25	0.71	0.18	0.02	2.90E-15	0.06
1	154426264	C	rs4129267	0.60	-0.02	0.16	0.91	0.59	0.16	0.02	1.22E-14	0.06
15	78563103	A	rs3169166	0.60	0.08	0.16	0.65	0.58	0.16	0.02	1.31E-14	0.06
6	31420500	T	rs3131622	0.59	0.06	0.16	0.70	0.55	-0.16	0.02	3.39E-14	0.05
14	23494277	A	rs8013143	0.72	-0.47	0.18	0.01	0.72	-0.17	0.02	4.59E-14	0.05
16	53820527	T	rs9939609	0.62	-0.35	0.16	0.03	0.61	-0.16	0.02	1.52E-13	0.05
11	16363658	C	rs11023922	0.93	0.10	0.31	0.75	0.92	-0.27	0.04	4.46E-13	0.05
5	1104938	C	rs35188965	0.45	0.01	0.16	0.94	0.42	0.15	0.02	5.38E-13	0.05
8	126486409	A	rs17321515	0.54	-0.31	0.16	0.06	0.53	-0.14	0.02	4.03E-12	0.04
12	121423956	C	rs2393791	0.39	-0.16	0.16	0.33	0.38	-0.15	0.02	7.37E-12	0.04
2	8750266	A	rs3856447	0.42	-0.06	0.16	0.73	0.40	-0.14	0.02	8.06E-12	0.04
6	135418635	C	rs7775698	-	-	-	-	0.74	0.16	0.02	9.59E-12	0.04
1	203652444	A	rs1419114	0.10	-0.33	0.26	0.20	0.10	-0.23	0.03	1.80E-11	0.04
12	51138687	A	rs2280503	0.67	-0.20	0.17	0.25	0.66	-0.14	0.02	3.07E-11	0.04
6	139840693	A	rs592423	0.45	0.26	0.16	0.11	0.45	0.14	0.02	3.38E-11	0.04
9	136131322	G	rs8176746	0.93	0.81	0.31	0.01	0.94	0.28	0.04	4.50E-11	0.04
18	43831259	A	rs9944715	0.28	-0.45	0.18	0.01	0.25	-0.16	0.02	6.04E-11	0.04
16	51436882	A	rs17616063	0.94	0.40	0.33	0.22	0.92	0.25	0.04	6.63E-11	0.04
6	32566149	G	rs9270664	0.38	-0.27	0.16	0.10	0.36	-0.14	0.02	6.65E-11	0.04
5	72406312	T	rs12657096	0.14	0.02	0.23	0.93	0.15	-0.19	0.03	7.55E-11	0.04
10	25207403	A	rs10828724	0.68	0.36	0.17	0.04	0.63	0.14	0.02	8.26E-11	0.04
19	18513594	T	rs1985157	0.61	-0.44	0.17	0.01	0.59	-0.14	0.02	1.09E-10	0.04
10	71093392	C	rs16926246	0.88	-0.36	0.25	0.15	0.87	-0.20	0.03	1.45E-10	0.04
2	113841030	A	rs6734238	0.61	-0.43	0.16	0.01	0.60	-0.13	0.02	1.49E-10	0.04
12	54649978	C	rs79880068	0.90	0.19	0.27	0.49	0.90	0.21	0.03	3.59E-10	0.04
14	65267469	T	rs230703	0.70	-0.14	0.18	0.43	0.71	-0.14	0.02	6.33E-10	0.04
11	100471466	A	rs11606890	0.91	0.37	0.27	0.17	0.90	0.21	0.03	6.87E-10	0.04
1	11856378	G	rs1801133	0.66	-0.07	0.17	0.68	0.66	-0.13	0.02	1.27E-09	0.03
19	35550878	C	rs45512696	0.85	0.32	0.23	0.15	0.82	0.16	0.03	1.45E-09	0.03
21	46257269	G	rs2838701	0.87	-0.05	0.24	0.84	0.87	-0.18	0.03	1.51E-09	0.03
2	27730940	T	rs1260326	0.40	0.08	0.16	0.62	0.39	-0.13	0.02	2.28E-09	0.03
17	76395430	C	rs2292642	0.39	0.07	0.16	0.67	0.40	-0.12	0.02	6.56E-09	0.03
16	28841777	C	rs12443881	0.61	0.04	0.16	0.83	0.60	-0.12	0.02	8.48E-09	0.03
17	31132529	T	rs17781005	0.81	0.12	0.21	0.56	0.81	0.15	0.03	9.08E-09	0.03
16	228306	G	rs8061637	0.94	-0.20	0.35	0.57	0.93	0.23	0.04	1.41E-08	0.03
5	67575344	A	rs16897620	0.77	-0.14	0.19	0.46	0.76	-0.14	0.02	1.90E-08	0.03
1	247601595	T	rs12239046	0.38	-0.29	0.16	0.08	0.37	-0.12	0.02	2.03E-08	0.03
10	45953767	A	rs7908745	0.68	-0.05	0.17	0.77	0.68	-0.12	0.02	2.91E-08	0.03
11	116648917	G	rs964184	0.16	0.36	0.22	0.10	0.13	-0.17	0.03	3.26E-08	0.03
20	31099311	T	rs159428	0.42	-0.27	0.17	0.11	0.47	-0.11	0.02	3.32E-08	0.03
17	27183104	G	rs9914988	0.23	-0.26	0.19	0.17	0.20	-0.14	0.03	4.24E-08	0.03
1	159683814	G	rs77832441	-	-	-	-	1.00	0.92	0.17	1.38E-07	0.03
10	46111895	G	rs74436700	0.98	-0.32	0.59	0.59	0.98	-0.36	0.07	1.93E-07	0.03

PVE: percent of the variance explained

Table S8 Replication results in HRS for the BioAgeAccel lead SNPs from UKB

				US Health Retirement Study				UK Biobank				
Chr	bp	rs	refA	freq	b	se	p	freq	b	se	p	PVE (%)
19	45412079	rs7412	C	0.92	0.00	0.13	0.98	0.92	0.26	0.02	0.00	0.27
19	11188153	rs55791371	A	0.89	0.16	0.11	0.16	0.88	0.14	0.01	0.00	0.11
7	106411858	rs17477177	T	0.80	-0.08	0.09	0.37	0.80	-0.09	0.01	0.00	0.07
2	21294975	rs541041	G	0.17	-0.11	0.10	0.23	0.18	-0.09	0.01	0.00	0.06
10	71093392	rs16926246	C	0.88	0.15	0.11	0.20	0.87	0.09	0.01	0.00	0.05
1	55505647	rs11591147	G	0.99	-0.11	0.35	0.75	0.98	0.23	0.03	0.00	0.05
8	126486409	rs17321515	A	0.54	0.10	0.07	0.16	0.53	0.06	0.01	0.00	0.05
6	32050544	rs3130287	C	0.12	-0.19	0.11	0.08	0.15	-0.08	0.01	0.00	0.05
19	19379549	rs58542926	C	0.92	0.11	0.13	0.40	0.92	0.11	0.02	0.00	0.05
1	21889760	rs149344982	G	0.99	0.81	0.50	0.10	0.99	0.25	0.04	0.00	0.04
2	169763148	rs560887	T	0.27	-0.03	0.08	0.67	0.30	-0.06	0.01	0.00	0.04
10	96039597	rs2274224	G	0.56	0.12	0.07	0.08	0.57	0.05	0.01	0.00	0.04
1	11862778	rs17367504	A	0.86	0.11	0.10	0.27	0.84	0.07	0.01	0.00	0.04
5	32815028	rs1173771	A	0.39	-0.06	0.07	0.43	0.40	-0.05	0.01	0.00	0.03
8	10635141	rs6601523	G	0.42	0.06	0.07	0.38	0.40	0.05	0.01	0.00	0.03
16	69965021	rs77870048	C	0.95	-0.04	0.18	0.85	0.95	-0.11	0.02	0.00	0.03
12	90060586	rs17249754	G	0.84	-0.03	0.10	0.73	0.83	0.07	0.01	0.00	0.03
20	10969030	rs1327235	A	0.55	-0.10	0.07	0.15	0.52	-0.05	0.01	0.00	0.03
15	91429176	rs7497304	G	0.70	0.11	0.08	0.14	0.67	-0.05	0.01	0.00	0.03
4	81184341	rs16998073	A	0.71	0.17	0.08	0.03	0.71	-0.05	0.01	0.00	0.03

PVE: percent of the variance explained

Table S9 Associations between PhenoAge or BioAge and *APOE* genotypes

PhenoAge*	n	Frequency	Estimate	Std. Error	2.50%	97.50%	P-Value
e3e3	3497	63.15%	reference	reference	reference	reference	reference
e2e2	38	0.69%	2.61	1.38	-0.08	5.31	0.058
e2e3	675	12.19%	0.38	0.36	-0.32	1.07	0.291
e2e4	107	1.93%	-1.71	0.83	-3.33	-0.08	0.039
e3e4	1122	20.26%	-0.56	0.29	-1.13	0.01	0.054
e4e4	99	1.79%	-0.10	0.86	-1.78	1.59	0.912
BioAge using 2016 biomarkers*	n	Frequency	Estimate	Std. Error	2.50%	97.50%	P-Value
e3e3	1104	62.44%	reference	reference	reference	reference	reference
e2e2	9	0.51%	-0.24	0.71	-1.63	1.15	0.733
e2e3	222	12.56%	0.05	0.16	-0.25	0.36	0.730
e2e4	44	2.49%	-0.10	0.32	-0.74	0.54	0.755
e3e4	362	20.48%	0.09	0.13	-0.16	0.34	0.473
e4e4	27	1.53%	-0.18	0.41	-0.99	0.63	0.661
BioAge using 2016 and 2014 biomarkers#	n	Frequency	Estimate	Std. Error	2.50%	97.50%	P-Value
e3e3	3,075	63.06%	reference	reference	reference	reference	reference
e2e2	32	0.66%	-0.06	0.37	-0.79	0.67	0.880
e2e3	598	12.26%	-0.03	0.09	-0.22	0.15	0.716
e2e4	96	1.97%	-0.14	0.22	-0.57	0.29	0.518
e3e4	989	20.28%	-0.01	0.08	-0.16	0.14	0.921
e4e4	86	1.76%	0.14	0.23	-0.31	0.59	0.551

*adjusted for age in 2016, sex, PC1-PC5

#adjusted for age in 2016, sex, PC1-PC5, and an indicator of using 2014 HbA1c and systolic blood pressure data

Table S10 ICD-10 disease codes

Disease	ICD-10 Codes	Notes
Age-Related Macular Degeneration	H353	
Anemia	D50-D53	
Anxiety	F40, F41	
Atrial Fibrillation	I48	
Bladder Cancer	C67	
Breast Cancer	C50	
Chronic Kidney Disease	N18; N183; N184; N185; Y841	
Colorectal Cancer	C18-20	
COPD	J42-J44	
Delirium	F05	
Dementia	F00; F01; F02; F03; G30	
Depression	F32; F33; F34.1	
Heart Failure	I50; J81	
Hypertension	I10-I15	
Hypothyroidism	E03	
Kidney Cancer	C64	
Liver Disease	K70-K77	Any
Lung Cancer	C34	
Melanoma Cancer	C43	Malignant Melanoma
Coronary Artery Disease	I20–I25	MI or Angina
Osteoarthritis	M15.0; M15.1; M15.2; M15.9; M16.0; M16.1; M17.0; M17.1; M18.0; M18.1; M19.0	
Osteoporosis	M80; M81; M81.1; M81.2; M81.3; M81.4; M81.5; M81.6; M81.8; M81.9	
Parkinson's Disease	G20; F02.3	
Peripheral Artery Disease	I70.2; I70.9; I73; I74.2; I74.3; I74.4; I74.5; I79.2	Peripheral Vascular Disease
Pneumonia	J13; J14; J15; J16; J17; J18	
Prostate Cancer	C61	
Renal Failure	N18; N18.0; N18.3; N18.4; N18.5; N18.8; N18.9	
Rheumatoid Arthritis	M05; M06	
Stoke	G45-G46; I61; I63	Stroke/TIA
Type I Diabetes	E10	
Type II Diabetes	E11	

Table S11 Parameters associated with each biomarker to derive BioAge

	Units	S	K	Q
Albumin	g/dL	0.3372441	-0.0057658	4.437681
Alkaline Phosphatase	u/L	28.91255	0.4822007	58.87995
Creatinine (Serum)	mg/dL	0.206529	0.0029099	0.9299351
CRP	mg/dL	0.6014597	0.0058188	0.1414078
Hba1c	%	0.9468073	0.0196594	4.488046
Systolic BP	mmHg	14.64641	0.6784407	90.98659
Total Cholesterol	mg/dL	39.93671	0.972077	163.2156
